# Levels of heavy metals in wastewater and soil samples from open drainage channels in Nairobi, Kenya: community health implication

**DOI:** 10.1038/s41598-020-65359-5

**Published:** 2020-05-21

**Authors:** Geoffrey K. Kinuthia, Veronica Ngure, Dunstone Beti, Reuben Lugalia, Agnes Wangila, Luna Kamau

**Affiliations:** 1 Daystar University, Department of Science, Engineering & Health, PO Box 44400-00100 GPO, Nairobi, Kenya; 2 Laikipia University, Department of Biological Sciences, PO Box 1100-20300, Nyahururu, Kenya; 3 Center for Virus Research – Kenya Medical Research Institute, PO Box 548840-00200, Nairobi, Kenya; 4 Kenyatta University, Department of Pharmacy and Complementary/Alternative Medicine, P.O. Box 43844-00100, Nairobi, Kenya; 5 Center for Biotechnology Research and Development (Malaria laboratory) – Kenya Medical Research Institute (KEMRI), PO Box 548840-00200, Nairobi, Kenya

**Keywords:** Environmental sciences, Risk factors

## Abstract

Levels of Mercury (Hg), Lead (Pb), Cadmium (Cd), Chromium (Cr), Nickel (Ni) & Thallium (Tl) were established in wastewater & soil samples obtained from 8 sites in open drainage channels at Nairobi industrial area, Kenya. Ultra-trace inductively coupled plasma mass spectroscopy (ICP-MS) was used for metal analysis. Temperature, pH & turbidity of wastewater ranged from 16.75 to 26.05 °C; 7.28 to 8.78; 160.33 to 544.69 ppm respectively and within World Health Organization (WHO) allowable limits. Wastewater conductivities in 4 sites ranged from 770 to 1074 *µ*S/cm and above WHO limits at 25 °C. The mean concentrations of the metals in wastewater ranged from 0.0001 to 0.015 ppm in an ascending order of Tl <Cd <Hg <Ni <Cr <Pb. Levels of Cd, Cr & Ni in wastewater were within WHO, World Bank (WB), United States Environmental Protection Agency (US EPA), China, Kenya and India’s Central Pollution Control Board (CPCB) limits while Hg & Tl were above US EPA limits. The mean Pb levels in wastewater (5 sites) were above WHO, US EPA and Kenya allowable limits. Mean levels of the metals in soil samples ranged from 0.085 to 199.99 ppm, with those of Hg, Pb, Cr, Cd & Ni being above WHO limits for agricultural soils. Positive correlations (P < 0.05) for Cd & Hg in soils; and Tl (wastewater) & Cd (soil) were noted. In conclusion, wastewater in open waste channels at Nairobi industrial area had elevated levels of Pb and Hg, while the soil from the same channels had high levels of Hg, Pb, Ni, Cr, and Cd. Good management of Nairobi industrial area effluents is inevitable since it borders densely populated informal settlements which are likely to suffer exposure to toxic wastes. Effective wastewater treatment and reuse is highly recommended.

## Introduction

Heavy metals are defined as metallic elements that have a relatively high density compared to water^1^. Heavy metals like Chromium (Cr), Cadmium (Cd), Mercury (Hg), Lead (Pb), Nickel (Ni), and Thallium (Tl) are potentially hazardous in combined or elemental forms. Heavy metals are highly soluble in the aquatic environments and therefore they can be absorbed easily by living organisms. Previous studies have detected heavy metals in the gills, liver, and muscles tissues of various species of fish in contaminated marine ecosystems^2^. Once the heavy metals enter the food chain, they may end up accumulating in the human body^3^. Since most heavy metals are widely applied in industries, exposure and contamination of the workers and residents near such facilities is likely to occur. Heavy metals above allowable limits will often lead to disadvantageous effects in humans, other organisms and the environment at large^4^. Allowable safe limits of heavy metals in food samples are associated with low health risks in humans^5,6^.

The level of toxicity of some selected metals for humans follows the sequence Co < Al < Cr < Pb < Ni < Zn < Cu < Cd < Hg^4^. The harmful effects of heavy metals in humans depends on their dosage, rate of emission and period of exposure. Some of the heavy metals that have received more attention for the last decades are Hg, Cd, and Pb^7^. The adverse health effects that are associated with Hg and mercuric compounds in humans includes possible carcinogens; damage of the brain, lungs and kidneys; damage of developing fetuses; high blood pressure or heart rate; vomiting and diarrhea; skin rashes and eye irritation^8^. The US EPA’s regulatory limit of Hg in drinking water is 2 parts per billion (ppb)^8^. The WHO recommended safe limits of Hg in wastewater and soils for agriculture are 0.001^9^ and 0.05 ppm respectively^10^.

Chronic toxicity of Cd in children includes damages of respiratory, renal, skeletal and cardiovascular systems as well as development of cancers of the lungs, kidneys, prostate and stomach^11,12^. Exposure of people to Cd includes, eating contaminated food, smoking cigarettes, and working in cadmium-contaminated work places and in primary metal industries^13^. A study carried out in Iran reported that the level of Cd was higher than the maximum permissible limit (MPL) in canned fish samples, and this was due to discharge of heavy metal rich pollutants into aquatic ecosystems^14^. The US EPA’s regulatory limit of Cd in drinking water is 5 ppb or 0.005 parts per million (ppm)^8^. The WHO recommended safe limits of Cd in both wastewater and soils for agriculture is 0.003 ppm^15,16^.

Exposure to Pb can occur through inhalation of contaminated dust particles and aerosols or by ingesting contaminated food and water. Lead poisoning in humans damages the kidneys, liver, heart, brain, skeleton and the nervous system^17^. Initial symptoms of poisoning associated with exposure to Lead may include headache, dullness, memory loss and being irritable^18^. Lead poisoning may cause disturbance of hemoglobin synthesis and anemia^19^. In children, chronic exposure to low levels of Lead may decrease their intelligence capacity. According to the International Agency for Research on Cancer (IARC), Lead is a possible carcinogenic substance in humans^19^. The regulatory limit of Pb in drinking water according to US EPA is 15 ppb^8^. The WHO recommended safe limits of Pb in wastewater and soils used for agriculture are 0.01 and 0.1 ppm respectively^15,20^.

Chromium is widely used in metallurgy, electroplating, and in the manufacturing of paints, pigments, preservatives, pulp and papers among others^21^. The introduction of Chromium into the environment is often through sewage and fertilizers^22^. Hexavalent Chromium compounds including chromates of Ca, Zn, Sr, and Pb are highly soluble in water, toxic and carcinogenic^21,23^. Furthermore, compounds of Chromium have been associated with slow healing ulcers. It has also been reported that Chromate compounds can destroy DNA in cells^24,25^. The WHO recommended safe limits for Cr (hexavalent) in wastewater and soils used for agriculture are 0.05 and 0.1 ppm respectively^15,16^.

Thallium is a soft, tasteless, odorless white blue metal in its pure form and it oxidizes to thallium oxide when exposed to air. Sources of Tl include electronics, optical glasses, semi-conductors, mercury lamp among others. Humans become exposed to Tl through ingestion, inhalation and dermal exposure. Thallium is highly toxic with a lethal dose of 6 to 40 mg/kg. Thallium poisoning is associated with anorexia, vomiting, gastrointestinal bleeding, abdominal pain, polyneuropathy, alopecia, renal failure, skin erythema, seizures, emotional changes, autonomic dysfunction, cardio toxicity, and coma among others^12^. In China, the recommended safe limit of thallium in drinking water is 0.0001 ppm^26^. The WHO recommended safe limits for Tl in both wastewater and agricultural soils were not given in the literature accessed.

Nickel is a silver - colored metal used in making stainless steel, electronics, and coins among other uses^27^. Globally, the release of Ni to the environment is estimated to vary from 150, 000 to 180, 000 metric tons per year^28^. Exposure of Ni to humans is through food, air and water^29^. Previous study has shown that ingestion of dust contaminated with Nickel was the main exposure pathway of the heavy metal by local residents when compared to inhalation and dermal pathways^30^. Upon exposure to Nickel, an individual may show increased levels of Ni in his or her tissues and urine. The disadvantageous effects of nickel on human health may include dermatitis, allergy, organ diseases, and cancer of the respiratory system^31^. The recommended safe limits by WHO for Ni in wastewater and agricultural soils are 0.02 and 0.05 ppm respectively^15,16^.

Wastewater from factories may contain heavy metals which with time accumulate in the soil deposits along waste water channels as well as in organisms that inhabit such channels. Exposure of humans to contaminated wastewater is often possible especially in urban highly populated areas or where the wastewater is reused for agricultural activities. Previous studies however have shown that effective reuse of wastewater is a major challenge in many countries of the world^32^.

The current study was designed to establish the concentration of Hg, Pb, Cr, Tl, Cd and Ni in samples of wastewater and soil obtained from open wastewater channels in selected sites in Nairobi’s industrial area, Kenya. There are many informal urban settlements/villages that neighbor Nairobi industrial area and some of the wastewater channels drain into a tributary of Nairobi river which flows across these villages. Clogged wastewater channels enhance overflow of the wastewater into the surrounding areas through surface runoff when it rains. Therefore, the current study aims to highlight the potential health risks that may ensue when humans, livestock and crops become directly or indirectly exposed to the heavy metal contaminated wastewater and soils from the open channels in the study area. It is envisaged that the results obtained from the study will inform and justify on the need to adopt good wastewater management including prioritizing on effective wastewater treatment and reuse in Kenyan major urban areas. Previous studies elsewhere have shown that the degree of wastewater treatment determines the applicability of the reclaimed water^33^.

## Methods and Materials

### Sampling sites

**Figure 1:**
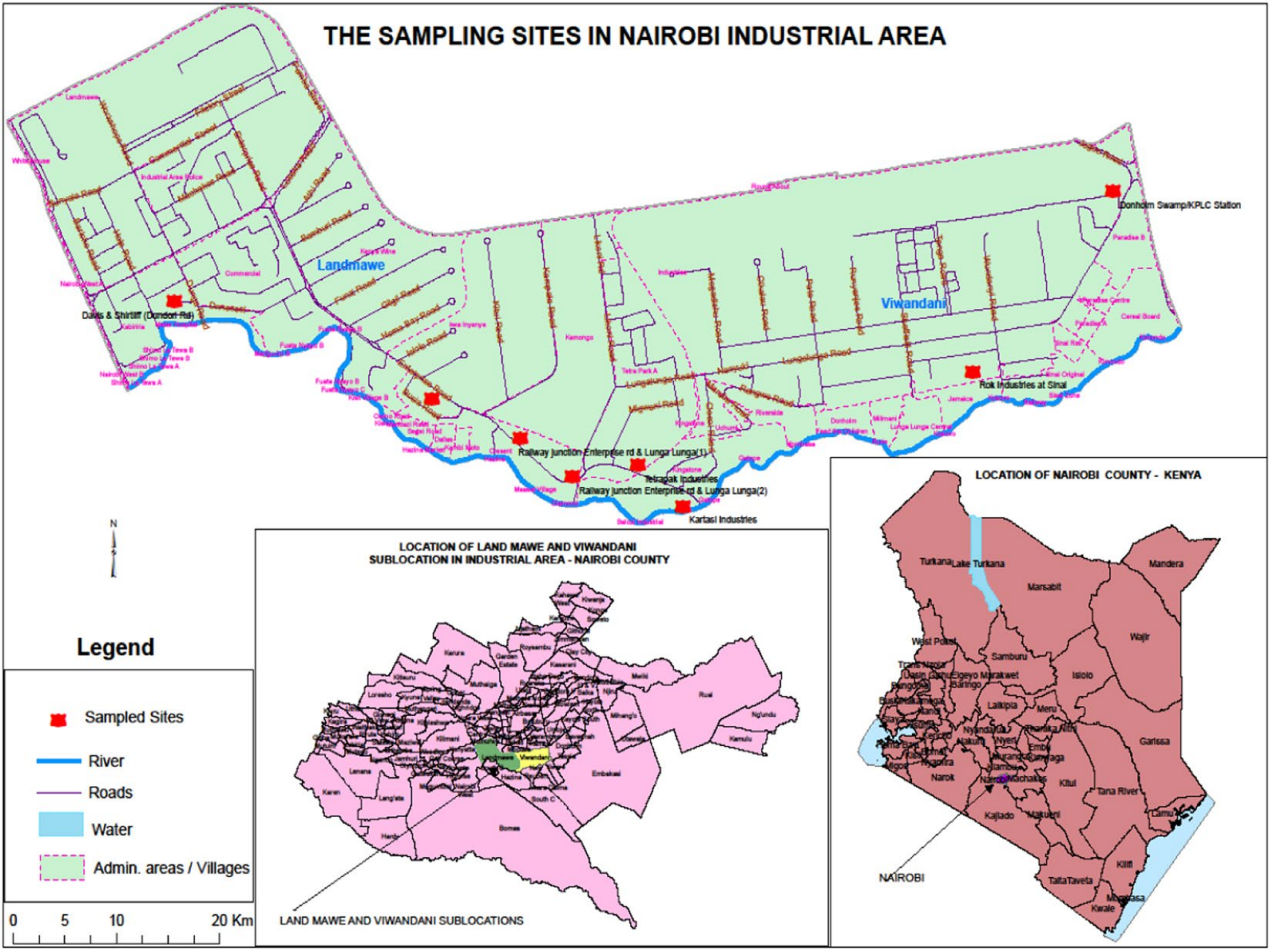
Showing the study area and the sampling sites in Nairobi industrial area in Kenya (Source: Kenya National Bureau of Statistics (KNBS); Software used to draw the map was ArcMap Version 10.61).

Samples were collected from Nairobi industrial area in Kenya from eight different sampling sites that were coded A to H. Figure 1 shows the sites which included Tetrapak (A); Chief’s Camp at Land Mawe (B); two sites at Railways near Enterprise/Lunga Lunga roads junction (C & D); Davis & Shirtliff company along Dondori road (E); Kartasi industries (F); Rok Industries-Sinai (G); and Donholm Swamp/Kenya Power and Lighting Station (H). The sampling sites were randomly selected along the main open wastewater channels on assumption that these were the channels that were consolidating the wastes from various directions in the industrial study area. From each site, samples of water and soils were collected from the open waste water channels. All the sampling sites were near informal settlements that included Sinai and Mukuru kwa Njenga urban slums/villages and also not far away from a tributary of Nairobi river; this decision was based on assumption that, the direct health implication of the findings on human population was likely to be established (Fig. 1).

### Collection of water and soil samples

A standard 350 ml dipper was used to collect samples of wastewater in triplicate portions, from open channels and put into clean reagent plastic bottles. Two separate portions of waste water destined for determination of heavy metals were acidified with concentrated hydrochloric acid (HCL) and concentrated nitric acid (HNO_3_) respectively to a pH ranging between 1.5 and 2.0 as described by Davies^34^. The third sample of waste water was left plain with no acidification. The samples were then labeled. Control samples of clean water were collected from tap water in randomly selected premises in the study area. Soil samples in triplicates were also collected from the open channels using a hand metallic soil scoop and then packaged into brown paper bags and labeled. The soil scoops were cleaned after every scooping was done. Both water and soil samples collected were immediately transferred to Kenyatta University Science Complex Laboratory for further processing.

### Physico - chemical parameters of water samples

Both physical and chemical parameters of the water samples were measured and recorded at the collection site. These included temperature, pH, electrical conductivity and turbidity. Electronic devices capable of recording the parameters at the same time (HANNA Instruments, H1991300, Romania) were used.

### Preparation of soil samples for heavy metal analysis

In the laboratory, the wet soil samples from each sampling site were spread on brown papers to dry under room temperature. They were then ground, sieved, weighed, and packaged in small brown envelops and labeled. The labels included site, date of collection, and weight in grams.

### Standard limits of heavy metals in waste water, drinking water, soils, and vegetables

The standard limits of Hg, Pb, Cd, Cr, Tl and Ni in drinking water, waste water (effluents), agricultural soils, and vegetables, as recommended by WHO; China (both Chinese Ministry of Health (CMH) & The National Standard of China); Kenya (both National Environment Management Authority (NEMA) & Kenya Bureau of Standards (KEBS); USA EPA; India (CPCB) and World Bank (WB) were retrieved from the available literature. The determined level of heavy metals in the field samples were then compared to these standard limits in order to establish whether the level of pollution in the open channels was above the locally and internationally accepted standards in addition to making reliable conclusions.

### Analysis of heavy metals in samples of water and soil

The analyses were carried out at Mineral Laboratories, Bureau Veritas Commodities Ltd, Vancouver, Canada. The protocols included *aqua regia* digestion ultra-trace inductively coupled plasma mass spectroscopy (ICP-MS) for soil samples; and ICP-MS (solutions >0.1% Total Dissolved Solids (TDS) for water samples as described by the American Herbal Products Association (AHPA)^35^. Briefly, the digest solution was nebulized and sample aerosols transferred to argon plasma. The high temperature plasma then produced ions, which were then introduced into the mass spectrometer, which then sorted out the ions according to their mass-to-charge ration. The ions were then quantified with an electron multiplier detector. Certificates of analysis and quality control reports for all the samples analyzed were awarded by the Bureau Veritas, Canada.

### Data analysis

Statistical Package for Social Sciences (SPSS) for Windows (Version 20) at 5% level of significance was used. Descriptive statistics involved computing the mean, standard error (SE), and standard deviation (SD) for the different variables measured in water and soil samples. One-way analysis of variance (ANOVA) was used to establish the significant differences within and between groups. Tukey’s and Games-Howell *Post hoc* tests were carried out to establish the pairs of variables that were significantly different. Correlation analysis was carried out to establish the nature of relationship, level of significance between concentrations of heavy metals in different samples. Pairwise correlation coefficients for the levels of selected heavy metals in waste water and soils were computed.

## Results

### Nature of waste water sampling sites

**Figure 2:**
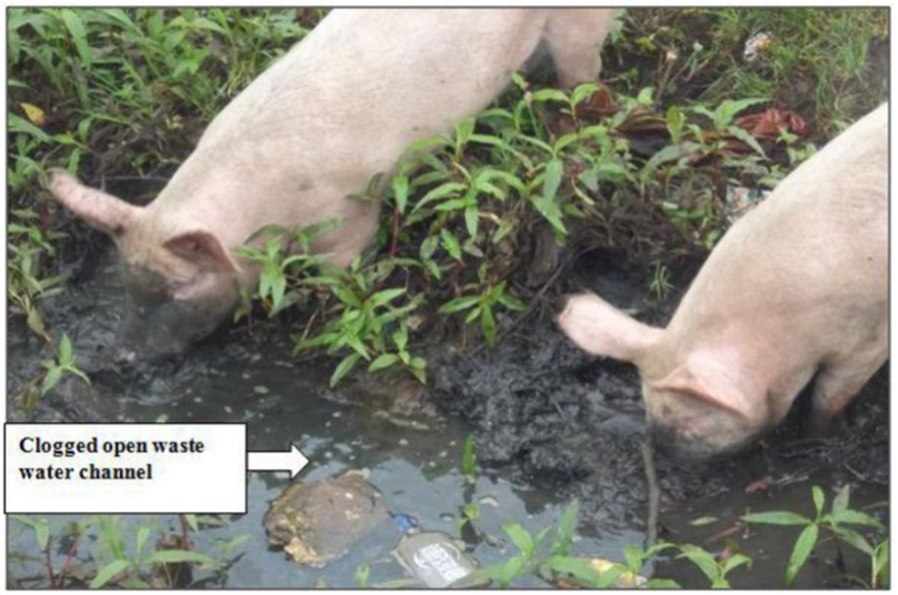
Showing two domestic pigs (*Sus scrofa domesticus)* scavenging for some edibles from the open waste water channel near the gate of Kartasi industry (F), Nairobi. Note: the open waste water channel was clogged with solid wastes (papers and plastic containers), mud and overgrown vegetation (The photograph was captured using a digital camera).

In 2 out of 8 (25%) open waste water channels, no overgrown vegetation was present and waste water was flowing effectively. In 5 out of 8 channels (62.5%), overgrown vegetation and/or trapped soil and mud was observed. In 1 out of 8 sites (12.5%), the Donholm site (H), it was an open swampy area with papyrus plants and stagnant brackish water. Domestic pigs were observed scavenging for edible materials from muddy waste channels clogged with overgrown vegetation and solid wastes at Kartasi sampling site F (Fig. 2).

### Physico-chemical parameters of waste water samples

The conductivity of waste water obtained from Chief’s Camp (B-2), Railways (sites C & D) and Sinai (G) was high and ranged between 770.00 ± 11.55 to 1134.33 ± 180.39 μS/cm (Table 1). The conductivity levels of waste water samples from the remaining sites ranged from 366.33 ± 33.79 to 556.00 ± 27.62 μS/cm. Increased conductivity corresponded to increased turbidity of waste water and vice versa. Therefore, high turbidity of waste water was similarly recorded at Railways upper section (D), Sinai (G) and Railways lower section (C) sites and it measured 535.33 ± 41.60, 544.67 ± 21.17 and 562.00 ± 84.33 ppm respectively. The chief’s camp (B-1) had the lowest turbidity at 160.33 ± 0.88 ppm (Table 1). The pH of wastewater samples ranged from 7.28 to 8.78 while the temperature ranged from 16.75 to 26.05 °C (Table 1). Waste water samples obtained from Chief’s camp (B-1), Kartasi industries (F) and Sinai (G) sampling sites were more alkaline compared to samples from other sites (Table 1). The temperature of waste water samples obtained from open, shallow and exposed channel at Sinai (G) site was high at 26.05 °C, while the temperature of waste water samples from sites that had vegetation cover and shade was lower, for example Davis & Shirtliff site (E), at temperature of 16.75 °C. The physico-chemical parameters recorded differed significantly (F-test, P < 0.05) as shown in Table 1.

**Table 1:** Showing the pH, temperature, turbidity, and conductivity of the waste water samples obtained from different sampling sites.

Site (code)	pH	Variables measured (± SE)		
		Temp (°C)	Turbidity (ppm)	Conductivity (*µ*S/cm)
Tetrapak (A)	7.51 ± 0.07	17.03 ± 0.12	230.67 ± 2.90	448.00 ± 11.02
Chief’s Camp (B-1)	8.13 ± 0.04	19.43 ± 0.04	160.33 ± 0.88	336.67 ± 8.82
Chief’s Camp (B-2)	7.64 ± 0.02	20.10 ± 0.12	412.00 ± 10.15	770.00 ± 11.55
Railways Lower (C)	7.28 ± 0.14	18.77 ± 0.38	562.00 ± 84.33	1134.33 ± 180.39
Railways Upper (D)	7.48 ± 0.02	19.20 ± 0.10	535.33 ± 41.60	1072.33 ± 89.76
Davis & Shirtliff (E)	7.57 ± 0.05	16.75 ± 0.07	202.00 ± 2.30	366.33 ± 33.79
Kartasi (F)	8.59 ± 0.23	18.35 ± 0.20	277.67 ± 13.13	556.00 ± 27.62
Sinai (G)	8.78 ± 0.09	26.05 ± 0.29	544.67 ± 21.17	1074.33 ± 59.61
Donholm (H)	7.69 ± 0.19	18.06 ± 0.41	213.00 ± 6.00	428.67 ± 8.57
**Range**	7.28–8.78	16.75–26.05	160.33–544.67	336.67–1134.33
**Significance level**	P < 0.05	P < 0.05	P < 0.05	P < 0.05

### Standard limits of heavy metals in drinking water, waste water, soils, and vegetables

The recommended limits of selected heavy metals according to WHO, Kenya (NEMA & KEBS), China (CMH & The National Standard of China), US EPA, India (CPCB) and World Bank were retrieved and recorded from the literature accessed (Table 2). The standard levels for Tl were not given for waste water, soils, and vegetables in the literature accessed. However, allowable level of Tl in drinking water, recommended by Chinese Ministry of Health and US EPA was available (Table 2). The Tl limit level in surface water as recommended by US EPA was also recorded (Table 2). Standards of heavy metals in agricultural soils in Kenya were missing in the literature accessed (Table 2). Other than being a reference in this study, Table 2 serves to consolidate the standard limits for easy access by other researchers and scholars.

**Table 2:** Showing limits of selected heavy metals in drinking water, waste water, soils and vegetables as recommended by WHO, Kenya, China, USA EPA, World Bank, and India (CPCB).

Organization /Country	The variable whose standards were reviewed	Recommended limits for the studied heavy metals (ppm)					
		Hg	Cd	Pb	Cr (hexavalent)	Tl	Ni
WHO	Drinking water^57^	0.006	0.005	0.01	0.1	NG	0.07
	Waste water (effluents)^16,20^	0.001^9^	0.003	0.01	0.05	NG	0.02
	Soils (for agriculture)^15^	–0.08^10^	0.003	0.1	0.1	NG	0.05
	Plants (Vegetables)^58^	0.1	0.02	0.1–0.3	1.3	NG	10
China (Chinese Ministry of Health; and The National Standards)	Drinking water^26^	0.0001	0.005	0.01	0.05	0.0001	0.03
	Waste water (effluents)^59^	0.005	0.03	1.0	0.5	NG	1.0
	Soils (for agriculture)^60,61^	0.3–1.0	0.3–0.6	80	150–300	NG	40–60
	Plants (vegetables)^62–65^	0.01	0.05–0.2	0.1–0.3	0.5–1.0	NG	1.0
Kenya (NEMA and KEBS)	Drinking Water^66,67^	0.02	0.01	0.05	NG	NG	NG
	Waste water (effluents)^66^	0.005	0.01	0.01	0.05	NG	0.3
	Public sewers^66^	0.05	0.5	1.0	0.05	NG	3.0
	Soils (for agriculture)	NG	NG	NG	NG	NG	NG
	Plants (Vegetables)^68^	0.01	0.05	0.3	NG	NG	NG
US EPA	Drinking Water^69,70^	0.002	0.005	0.05	0.1 (total Cr)	0.013	0.02
	^a^SWQS limit level^71^	0.002^72^	0.009	8.5	0.08	6.3	8.3
	Water reclaimed from effluent for irrigation^73^	NG	0.01	5.0	0.1	NG	0.2
	Waste water (effluents)^74^	0.00003	0.01	0.006	0.05	NG	0.2
	Soils (for a garden)^75^	1.0	0.48	200	11	NG	72
	Plants (Vegetables)^76^	0.015	0.2	0.3^77^	2.3^77^	NG	NG
World Bank	Waste water (effluents)^78^	0.01	0.1	0.1	0.5 (total Cr)	NG	0.5^10^
India CPCB	Inland surface water^79^	0.01	2.0	0.1	0.1	NG	3.0
	Public sewers^79^	0.01	1.0	1.0	2.0	NG	3.0

^a^SWQS stands for Surface Water Quality Standards; The references for the recommended limits (ppm) are shown in superscript; ppm implies mg/kg or mg/L; NG stands for Not Given.

### Levels of heavy metals in waste water and tap water

The waste water samples had high Pb levels followed by Cr and the lowest was Tl. The mean concentration of heavy metals analyzed in waste water samples, in an ascending order was Tl < Cd < Hg < Ni < Cr < Pb. This trend applied for all samples of waste water that were acidified immediately after collection. The mean concentration of chromium was the highest at 24.2 ppb followed by nickel at 2.90 ppb in the waste water samples that were not digested by acids immediately after collection hence an ascending order of Tl < Cd < Hg < Pb < Ni < Cr.

The mean concentration of Hg in the waste water samples was <0.0001 ppm and this was lower than the standards set by WHO, World Bank (WB), Kenya, India and China but greater than 0.00003 ppm which is the standard set by US EPA (Tables 2, 3, 4). The level of Hg in waste water samples from all the sampling sites was below the method detection limit (MDL) which had been set at 0.1 ppb. Similarly, the average levels of Pb for acid digested waste water samples in 5 out of 8 (62.5%) sites (2 sites at Chief’s camp; Davis & Shirtliff, Kartasi and Donholm) had high Pb levels that ranged from 0.011 to 0.032 ppm (Tables 3 and 4), and this was above the recommended limits of Pb in waste water set by WHO, Kenya, and US-EPA (Table 2).

**Table 3:** Showing the levels (ppb or micrograms per liter) of heavy metals (Hg, Pb, Cr, Cd, Tl, and Ni) for the HNO_3_ digested. water samples (waste and clean) obtained from different sites.

Site of samples collection	Sample code	Heavy metals analyzed (ppb or *µ*g/L)					
		Hg (0.1)^a^	Pb (0.1)	Cr (0.5)	Cd (0.05)	Tl (0.01)	Ni (0.2)
**Waste water:**							
Tetrapak	A2	<0.1	9.5	0.7	0.09	<0.01	1.7
Chief’s Camp	B3a	<0.1	29.5	2.9	0.08	0.04	2.1
Chief’s Camp	B3b	<0.1	28.2	2.7	0.11	0.09	2.6
Railways Lower	C2	<0.1	8.3	9.8	0.17	0.08	21.1
Railways Upper	D2	<0.1	6.9	2.1	0.09	0.08	6.1
Davis & Shirtliff	E2	<0.1	24.2	1.9	0.13	0.05	2.6
Kartasi	F3	<0.1	17.3	1.4	0.08	0.03	6.5
Sinai	G3	<0.1	0.4	50.7	<0.05	0.03	<0.2
Donholm	H2	<0.1	13.5	0.9	<0.05	0.02	1.7
Mean concentration ± SE (ppb)		<0.1 ± 0.00	15.31 ± 3.39	8.12 ± 5.40	0.09 ± 0.01	0.05 ± 0.009	4.96 ± 2.13
Mean concentration (ppm or mg/l)		<0.0001	0.01531	0.00812	0.00009	0.00005	0.00496
**Tap water (control) - ppb:**							
Roysambu (House)^b^	I3	< 0.1	1.6	0.6	<0.05	<0.01	<0.2

^a^The value in bracket shows the method detection limit (MDL) measured in ppb. ^b^Tap water samples were acidified with HNO_3_ before metal analysis.

**Table 4:** Showing the levels (ppb or micrograms per liter) of heavy metals (Hg, Pb, Cr, Cd, Tl, and Ni) for the HCl digested waste water samples obtained from different sites.

Site of samples collection	Sample code	Heavy metals analyzed (ppb or *µ*g/L)					
		Hg (0.1)^a^	Pb (0.1)	Cr (0.5)	Cd (0.05)	Tl (0.01)	Ni (0.2)
**Waste water:**							
Tetrapak	A3	<0.1	9.1	0.8	0.08	0.02	2.2
Chief’s Camp	B2a	<0.1	31.5	2.8	0.12	0.04	2.4
Chief’s Camp	B2b	<0.1	24.1	2.3	0.14	0.09	2.6
Rialways Lower	C3	<0.1	8.5	7.8	0.11	0.07	22.3
Railways Upper	D3	<0.1	5.4	2.2	0.15	0.08	6.3
Davis & Shirtliff	E1	<0.1	14.5	1.2	0.07	0.04	2.2
Kartasi	F2	<0.1	10.6	1.1	0.07	0.04	3.6
Sinai	G1	0.1	6.2	8.5	0.27	0.09	19.4
Donholm	H3	<0.1	12.7	1.0	<0.05	0.01	1.2
Mean concentration ± SE (ppb)		< 0.1 ± 0.00	13.62 ± 2.91	3.08 ± 0.99	0.12 ± 0.02	0.05 ± 0.01	6.91 ± 2.69
Mean concentration (ppm or mg/l)		< 0.0001	0.01362	0.00308	0.00012	0.00005	0.00691
**Tap water (Control) - ppb:**							
Hotel (study area)	I1^b^	< 0.1	<0.1	1.6	<0.05	<0.01	<0.2
Chief’s camp (House)	I2	< 0.1	1.2	0.7	<0.05	<0.01	<0.2

^**a**^The value in bracket shows the method detection limit (MDL) measured in ppb. ^b^Tap water sample I1 was not acidified before metal analysis while sample I2 was acidified with HCl before metal analysis.

The mean concentration of Cr in waste water samples from all the sampling sites ranged between 0.00308 to 0.00812 ppm (Tables 3 and 4) which was between 84% to 99% less than the recommended limits by WHO, China, Kenya, US EPA, WB and India. The wastewater samples collected at Sinai (G1) had the highest concentration of chromium at 0.0507 ppm but which was within the maximum limit recommended level set by WHO and US EPA (Tables 2 and 3).

The mean concentration of Ni in waste water in all sampling sites was 0.004ppm and this was within the recommended limits set by WHO, China, Kenya, US EPA, WB and India (Table 2). Nickel level was significantly high in wastewater samples obtained from Railways Lower (C2) with a mean concentration of 21.7 ppb and at Sinai (G1) with a concentration of 19.4 ppb (Tables 3 and 4). The mean levels of Tl in the waste water was about 100 000 times less than the US EPA (SWQS) recommended limits (Table 2). The mean concentration of Cd in waste water in all the sampling sites was 0.000087 ppm which was far less than the recommended limits by WHO, WB, US EPA, China, Kenya, and India (Tables 2 to 4).

The level of Hg in samples of tap water was below the MDL which had been set at 0.1 ppb or 0.0001 ppm (Tables 3 and 4). Similarly, the levels of Pb, Cd, Cr, Tl and Ni in the samples of tap water ranged between 0.00001 and 0.0016 mg/ml (ppm) which were far below the standard limits set by WHO, Chinese Ministry of Health and Kenya (NEMA), US EPA (Tables 2, 3 and 4).

### The **levels of heavy metals in soil samples**

The mean concentration ± SE (standard error) of heavy metals in soil samples was highest for Pb and lowest for Hg in an ascending sequence of Hg < Tl < Cd < Ni < Cr < Pb (Table 5). The concentration of Pb in soil samples from Davis & Shirtliff site was 471.17 ± 117.5 ppm compared to samples collected from Chief’s Camp (B) and Railways Lower (C) sites that were at 255.50 ± 91.20 and 211.00 ± 8.26 ppm respectively. Soil samples from Sinai site had the lowest level of Pb at 59.92 ± 8.42 ppm. Relatively higher levels of Hg were recorded for soil samples collected at Chief’s Camp (B), Railways Lower (C), and Davis & Shirtliff (E), which were at 223.75, 121.00, and 106.67 ppb respectively (Table 5). The concentration of Cd and Tl in the soil samples ranged from 0.2 ± 0.05 to 1.90 ± 1.40 ppm and 0.23 ± 0.01 to 0.50 ± 0.06 ppm respectively (Table 5). Soil samples from Chief’s Camp (B) site had the highest level of Cd and Tl while samples from Donholm (H) site had the lowest (Table 5). The concentration of Cr and Ni ranged between 21.37 ± 9.87 to 81.17 ± 3.80 and 11.70 ± 0.44 to 29.87 ± 1.90 ppm respectively for the soil samples obtained from the study area (Table 5).

**Table 5:** Showing the mean ± SE levels (ppm) of heavy metals (Hg, Pb, Cr, Cd, Tl, and Ni) for soil samples collected in triplicates (except at site B) from eight different sites in Nairobi industrial area, Kenya.

Site of samples collection	Samples codes	Heavy metals analyzed (ppm or ppb)					
		Hg (5)^a^	Pb (0.01)	Cr (0.5)	Cd (0.01)	Tl (0.02)	Ni (0.1)
Tetrapak	A1, A2, A3	52.33 ± 4.33	118.28 ± 9.46	22.40 ± 2.09	0.61 ± 0.07	0.34 ± 0.01	15.50 ± 0.64
Chief’s Camp	B1, B2, B3, B4	223.75 ± 63.90	255.50 ± 91.20	31.68 ± 11.11	1.90 ± 1.40	0.50 ± 0.06	17.03 ± 3.61
Railways Lower	C1, C2, C3	121.00 ± 8.26	211.00 ± 8.26	81.17 ± 3.80	1.03 ± 0.05	0.35 ± 0.02	26.53 ± 0.77
Railways Upper	D1, D2, D3	75.00 ± 25.03	165.96 ± 7.14	67.83 ± 6.66	1.58 ± 0.82	0.43 ± 0.01	29.87 ± 1.90
Davis & Shirtliff	E1, E2, E3	106.67 ± 8.25	471.17 ± 117.5	72.80 ± 23.36	0.82 ± 0.05	0.47 ± 0.02	17.50 ± 0.26
Kartasi	F1, F2, F3	56.33 ± 12.20	174.48 ± 8.48	36.70 ± 3.37	0.53 ± 0.08	0.31 ± 0.01	16.30 ± 0.67
Sinai	G1, G2 G3	23.67 ± 3.38	59.92 ± 8.42	27.63 ± 5.55	0.31 ± 0.01	0.43 ± 0.06	16.07 ± 0.07
Donholm	H1, H2, H3	26.00 ± 2.31	143.56 ± 62.17	21.37 ± 9.87	0.20 ± 0.05	0.23 ± 0.01	11.70 ± 0.44
Mean concentration (ppm)		85.59 ± 23.24^b^	199.99 ± 43.92	45.19 ± 8.68	0.87 ± 0.21	0.38 ± 0.03	18.81 ± 2.16

^**a**^The value in bracket shows the method detection limit (MDL). All the units were ppm except for Hg which was in ppb; ^b^The mean concentration for Hg is in ppb units.

When compared to the standard limits, the mean concentration of Hg and Ni in soil samples was 0.085 and 18.81 ppm respectively (Table 5) and this was below the recommended limits set by China and US-EPA but above WHO limits for agricultural and gardening soils (Table 2). The mean concentration of Cr in the soil samples was 45.19 ppm and it was above the limits set by WHO and US EPA (Tables 2 & 5). The average levels of Pb and Cd in soil samples was also above the recommended limits set by WHO, China but within the US EPA limit for agricultural and gardening soils. The mean concentration of Tl in the soil samples was 0.38 ppm, however the soils standards for Tl in agricultural soils for WHO, US EPA, China and Kenya were not available. It was observed that the mean concentration of heavy metals analyzed in the soil samples was between 800 to 13,500 times more than the mean concentration of the same metals in waste water samples at the same sampling site (Tables 3–5).

### Correlation of heavy metals level in different samples

Pairwise comparison of the level of heavy metals in waste water and soils samples yielded several strong significant positive correlations (P < 0.05). Such pairs included Cd & Hg in soils samples; and Tl (waste water) & Cd (soil); where an increase in one element corresponded to an increase in the other (Table 6). The strong positive correlations indicated a close association of the elements in samples of soil and waste water. A negative correlation between Tl (soil) & Cr (waste water), r = − 0.641, P = 0.087, was also observed (Table 6).

**Table 6:** Inter-elemental correlation of heavy metals in samples of waste water and soils that were significant or nearly significant.

Pairs correlated	Correlation co-efficient (r - value)	P value
Cd (soil^a^) & Hg (soil)	0.839	0.009**
Tl (waste water) & Cd (soil)	0.967	0.000**
Tl (waste water) & Cd (waste water)	0.631	0.069
Tl (soil) & Cr (waste water)	−0.641	0.087
Tl (soil) & Cd (soil)	0.672	0.068

^a^Type of sample analyzed is placed in brackets; *correlation significance at 0.05 level (2 tailed); **correlation significance at 0.01 level (2 tailed).

At Sinai sampling site (G), Inter-elemental correlation levels of metals for the samples of waste water and soils samples were computed (Table 7). A significant positive correlation was obtained for pairs Cd & Pb; Ni & Pb in samples of waste water (Table 7). Similarly, a strong positive correlation coefficient (r = 0.995) for Cr & Pb that was near significance level (P = 0.061), was recorded for soil samples obtained from Sinai site (Table 7).

**Table 7:** Inter-elemental correlation coefficients (r-value) of heavy metals in samples of wastewater and soils obtained from Sinai sampling site (G) in the study area.

	Hg	Pb	Cr	Cd	Tl	Ni
**Wastewater samples:**						
Hg	−^a^					
Pb	—	1				
Cr	—	−0.820	1			
Cd	—	0.998*	−0.784	1		
Tl	—	0.453	−0.881	0.397	1	
Ni	—	0.998*	−0.858	0.992	0.513	1
**Soil samples:**						
Hg	1					
Pb	0.597	1				
Cr	0.517	0.995	1			
Cd	0.893	0.894	0.847	1		
Tl	0.071	−0.759	−0.817	−0.386	1	
Ni	−0.985	0.451	0.364	−0.803	−0.240	1

^a^Implies that correlation could not be computed because one of the variables was constant (the level of Hg was <0.1 ppb throughout); *Correlation significance at the 0.05 level (2 tailed).

## Discussion

Conductivity is a measure of the water’s ability to conduct electricity and it is directly related to the concentration of ions in the water. Significant changes in the conductivity of water directly compromise its quality. In the current study, conductivity of the waste water was highest for samples obtained from Railways lower (C), Railways upper (D) and Sinai (G) sampling sites and it ranged from 1072 to 1134 *μ*S/cm (Table 1). This range was higher than WHO range for electrical conductivity of water which is between 400 to 600 μS/cm^36^. The waste water samples from the remaining sites had conductivity levels that were within the WHO standards (Table 1). The source of the conductive ions in waste water may have been the dissolved substances including pollutants. At railways sampling sites (C and D), combined organic sewage material overflow that had been directed into the open waste water channel may have introduced dissolved ions into the channels hence increasing conductivity. This was in line with a previous study by Mbui and colleagues who reported that domestic effluents discharge into the river increases the electrical conductivity^37^. The road construction activities near Sinai sampling site (G) which involved heavy machinery plus nearby industries may have contributed to increased particles and ions in the waste water channels hence accounting for the raised turbidity and conductivity levels of the waste water samples. It was noted that increased turbidity corresponded to increased conductivity and vice versa (Table 1). Turbidity indicates total suspended solids in water and it is a principle parameter of waste water effluent monitoring and therefore it can be used to evaluate waste water treatment plant efficiency and compliance to discharge requirements^38^. Both conductivity and turbidity are important parameters in measuring the quality of water post treatment.

The temperature of waste water samples ranged from 16.75 to 26.05 °C (Table 1), which was within the recommended WHO range of 20 to 32 °C^9^. The pH of waste water samples ranged from 7.28 to 8.78 and similarly was within WHO range of 6.5 to 8.5^36^. Microbial degradative activities in waste water are dependent on temperature, pH, presence of organic substances and types of microbes. Therefore, elevated temperature in waste water tends to support increased bio-degradative reactions in presence of increased organic substances. The organic substances and types of microbes in the waste water were not determined in this study.

During the third United Nations Environment Assembly hosted by Kenya at UNEP headquarters (Gigiri) in December 2017, Kenya promised to improve the lives of its people by cleaning up air, land and water. Industrial, sewage and domestic wastes have been finding their way into Nairobi river in Kenya, hence making the river unsuitable for use^39^. According to UN Environment, over 80% of the world’s waste water is released into the environment without treatment, polluting the fields where plants grow, lakes and rivers^39^. Such pollutants can easily flow from the environment into humans directly or indirectly. Water and soil pollution with heavy metals in developing countries emanate from poor disposal of industrial and urban wastes^40^. Municipal and industrial wastewater should be treated as a strategy of minimizing the contaminants before reusing wastewater. However, health impact assessment of treated wastewater should be carried out to identify the hazards and risk factors that may be associated with wastewater reuse^41^.

The current study established that the levels of Cr, Cd, and Ni in waste water were below the limits set by WHO, US EPA, WB and Kenya. Similarly, the Hg level in waste water was below the limits set by WHO, China, WB, India and Kenya (NEMA) but slightly above the limit set by US EPA. The level of Pb in the waste water was above limits set by WHO, US EPA, WB and Kenya but lower than the limits set by China and India. The public health concern in terms of waste water in Nairobi industrial area is therefore Hg and Pb levels which were above the limits set by US EPA and WHO respectively. Standards for thallium in waste water were not available and therefore it was difficult to make a conclusion on whether the levels obtained were high or low. Previous reports however, have shown that water quality within Nairobi river catchment area in Kenya has degraded due to intensive land use hence increasing Hg and Pb levels and surpassing the critical guidelines of WHO^42^. In the current study area, humans can become exposed to such pollutants when surface runoff find its way into residential areas or utilizing the contaminated soils for agricultural activities. Heavy metal accumulation in soils leads to increased phyto - accumulation of such metals in the crops grown^40^. Clogging of open waste water channels with solid wastes, mud and overgrown vegetation can enhance surface runoff of the wastewater to surrounding areas. One of the factors which may increase the chance of exposure to metal pollutants in the study area is presence of dense population in the informal settlements near Nairobi industrial area. Some of the open waste water channels pass through these settlements or drain into Nairobi river which then flow across these settlements. However, treatment of wastewater for reuse is a common practice in many countries since it can alleviate natural water shortage and minimize contaminants finding their way into natural aquatic ecosystems^43^. According to a study carried out in Greece, the annual percent contribution of treated wastewater in the total irrigation water volume in Thermos and Nafpaktos was 87.8% and >100% respectively^44^. The same should be  adopted in Nairobi to minimize the contaminants in wastewater and to provide adequate water for agricultural activities.

Tap water samples (controls) that were randomly collected from the study area were analyzed to establish the heavy metals levels and compared to waste water samples. The levels of all the heavy metals studied in tap water samples ranged from <0.00001 to 0.0016 ppm (Tables 3 and 4) and these levels were far below limits set by WHO, US EPA, Chinese Ministry of Health (CMH) and Kenya (NEMA). The tap water sampled from residential and hotels in study area was therefore safe, high quality, and acceptable in terms of Hg, Pb, Cd, Cr, Tl and Ni levels.

The domestic pigs observed at Kartasi industries sampling site, scavenging for edibles from the mud and vegetation clogged open waste water channels (Fig. 2), was an evidence that there were residential areas nearby in the study area. The sampling sites in the current study were actually near the densely populated informal settlements (slums) that included Sinai, Mukuru kwa Njenga, and Land Mawe. The samples of waste water and soil sediments from Kartasi sampling site had levels of heavy metals that were above the MDL except for Hg level in waste water (Tables 3, 4, and 5). It is worth noting that even low levels of environmental metal pollutants can accumulate with time in exposed humans and animals. Previous studies show that livestock are prone to general problems of industrial pollution^45^. A study in Namibia established that pasture grass that was obtained from around waste dumpsites had higher levels of heavy metals^46^. It is possible for heavy metals to accumulate in the tissues and organs of domestic animals that become exposed to contaminated environments, materials and fodder. The concentration of Hg and Cd was shown to be high in the liver, kidney and muscle samples of organically and conventionally produced pigs in Czech Republic^47^. Therefore, the scavenging pigs in the open waste water channels in industrial area, that were observed in the current study, can serve to directly or indirectly spread the heavy metal pollutants from such channels into humans. When the heavy metals pollutants from the channels accumulate in the pigs’ muscles with time, then the quality of pork from such animals is compromised and it may become a health risk

Heavy metals occur naturally in soils following the weathering processes of the underlying rocks^48^. Availability of heavy metals in soils is influenced by environmental conditions that determine the pH and organic matter content in soils^49^. Heavy metal contamination of the soils may pose risks and hazards to humans and ecosystems through direct contact or ingestion, food chain, contaminated drinking water, reduced food quality among others^49^. The concentration of the Lead (Pb) in the soil samples at Davis & Shirtliff sampling site was 471.17 ± 117.5 ppm and this was above the normal range of Pb (2 to 300 ppm) in the soils^50–52^. The worldwide Pb concentration for surface soil averages 32 mg/kg (ppm) and it ranges from 10 to 67 mg/kg (ppm)^53^ implying that the levels at Davis & Shirtliff were significantly above this limit. The average Pb level in the soil samples collected from Chief’s camp (B) and Railways Lower (C) were 255.50 ± 91.20 and 211.00 ± 8.26 ppm respectively and they were skewing towards the upper limit of normal range as described by Gardea-Torresdey and colleagues^51^ but above the range reported by Pendias & Pendias^53^. The soil samples from Sinai site had the lowest level of Pb at 59.92 ± 8.42 ppm which was skewed towards the lower limit of normal range of Pb in soils. Soil sediments in the waste water channels may enrich with pollutants present in waste water with time. Increased Pb content in soils recovered from the open waste water channels is a health hazard to workers who regularly clean up the channels especially when they are ignorant about the need to maximize on safety measures. Lead has been associated with multiple organ problems and cancers. The soil samples collected from Chief’s camp (B), Railways Lower (C), and Davis & Shirtliff (E) sampling sites had relatively higher Hg levels but which were within the limits set by China and US EPA for agricultural soils. The average concentration of Cr and Ni in the soil samples from the study area which ranged between 21.37 ± 9.87 to 81.17 ± 3.80 and 11.70 ± 0.44 to 29.87 ± 1.90 ppm respectively were below the allowable limits recommended by China and US EPA but above the limits recommended by WHO for agricultural soils (Tables 2 and 5). It is important to note that even soils that are contaminated with low levels of heavy metals can contribute to bioaccumulation of such elements with time in organisms that are in higher trophic levels in a food chain. Pollution of environment with traces of heavy metals from anthropogenic sources should not therefore be ignored.

The mean concentration of heavy metals was higher in soils than in waste water samples. This was in line with a previous report by Khan and his colleagues^54^ which explained that contaminated wastewater can lead to a build-up of heavy metals in soils. Inter-elemental analysis of the metals showed several strong and positive correlations (Tables 6 and 7). This suggested that, these metals were from the same source, most likely the industries whose wastes were draining into the open channels in the study area. This explanation was in line with previous studies carried out in Nigeria and Pakistan^55,56^. The significant correlation coefficients between pairs of metals in samples of waste water and soils may be a pointer of a common source of the heavy metal pollution in the study area, most likely anthropogenic activities.

## Conclusion and recommendation

This study showed that wastewater and soils samples from open waste channels in Nairobi industrial area contained heavy metals. Of the metals studied, the mean concentration of Ni, Cr and Pb were relatively higher than those of Tl, Hg, and Cd in the samples analyzed. The levels of Hg, Cr, Cd and Ni in wastewater samples were within the allowable limits set by WHO, WB, Kenya, China and India. The mean level of Hg in wastewater was <0.0001ppm and this was a public health concern in the study area, based on the US EPA allowable limit of Hg in wastewater that is set at 0.00003 ppm. The level of Tl in wastewater samples was below the limit set by US EPA and this was commendable. The mean concentration of Pb in wastewater was above the allowable limits set by WHO, WB, US EPA and Kenya in 5 out of 8 sampling sites, hence becoming a public health concern in the study area. The levels of Pb, Hg, Cr, Cd, and Ni in open drainage channels soil samples were above the limits set by WHO for agricultural and gardening soils. The mean concentration of heavy metals was relatively higher in soil than in wastewater samples at each sampling site. This was an evidence of a build-up of toxic metals in the soils found in open waste channels. There was adequate evidence of clogging of the wastewater channels with mud and overgrown vegetation hence facilitating overflow and spread of contaminated wastewater and soils from the channels to residential areas nearby during the rainy seasons. Presence of domestic pigs scavenging from the open channels suggested a likely pathway through which the metallic contaminants could eventually find their way into humans. Therefore, there is need to formulate and adopt policies, strict rules among others that would translate to excellent wastewater management and treatment infrastructure hence minimizing environmental pollution and its associated health hazards as well as avail adequate reclaimed water for urban agricultural activities. Frequent inspections and unclogging of the open waste channels should be carried out to enhance faster flow and to minimize possible spread of heavy metal contaminated wastewater to the densely populated informal settlements/villages that neighbor Nairobi industrial area. Residents living nearby should be made aware of the health hazards that could emanate from exposure to untreated wastewater through public education and awareness campaigns. We the authors recommend the determination of heavy metals in pork available in the study area in order to provide possible evidence of bio-accumulation of these metal contaminants in human food.

## Data Availability

The datasets generated and/or analyzed during the current study are not publicly available but are available from the corresponding author on reasonable request.
