# Publisher Correction: Levels of heavy metals in wastewater and soil samples from open drainage channels in Nairobi, Kenya: community health implication

**DOI:** 10.1038/s41598-020-68286-7

**Published:** 2020-07-07

**Authors:** Geoffrey K. Kinuthia, Veronica Ngure, Dunstone Beti, Reuben Lugalia, Agnes Wangila, Luna Kamau

**Affiliations:** 1 Daystar University, Department of Science, Engineering & Health, PO Box 44400-00100 GPO, Nairobi, Kenya; 2 Laikipia University, Department of Biological Sciences, PO Box 1100-20300, Nyahururu, Kenya; 3 Center for Virus Research − Kenya Medical Research Institute, P.O. Box 548840-00200, Nairobi, Kenya; 4 Kenyatta University, Department of Pharmacy and Complementary/Alternative Medicine, P.O. Box 43844-00100, Nairobi, Kenya; 5 Center for Biotechnology Research and Development (Malaria Laboratory) − Kenya Medical Research Institute (KEMRI), PO Box 548840-00200, Nairobi, Kenya

Correction to: *Scientific Reports* 10.1038/s41598-020-65359-5, published online 21 May 2020

The Article contains a typographical error in Table 2 where the WHO recommended limit for Hg in Soils (for agriculture) was incorrectly given as “– 0.08” ppm. The correct recommended limit should read as “0.05–0.08” ppm.

